# Non-pharmacological interventions to improve sleep quality and quantity for hospitalized adult patients—co-produced study with surgical patient partners: systematic review

**DOI:** 10.1093/bjsopen/zrae018

**Published:** 2024-04-10

**Authors:** Radhika Acharya, Sue Blackwell, Joana Simoes, Benjamin Harris, Lesley Booth, Aneel Bhangu, James Glasbey

**Affiliations:** National Institute of Health and Care Research (NIHR) Global Health Research Unit on Global Surgery, University of Birmingham, Institute of Translation Medicine, Birmingham, UK; Patient Liaison Group (PLG), Association of Coloproctology of Great Britain and Ireland, London, UK; National Institute of Health and Care Research (NIHR) Global Health Research Unit on Global Surgery, University of Birmingham, Institute of Translation Medicine, Birmingham, UK; National Institute of Health and Care Research (NIHR) Global Health Research Unit on Global Surgery, University of Birmingham, Institute of Translation Medicine, Birmingham, UK; Patients and Researchers Together (PART), Bowel Research UK, London, UK; National Institute of Health and Care Research (NIHR) Global Health Research Unit on Global Surgery, University of Birmingham, Institute of Translation Medicine, Birmingham, UK; National Institute of Health and Care Research (NIHR) Global Health Research Unit on Global Surgery, University of Birmingham, Institute of Translation Medicine, Birmingham, UK

## Abstract

**Background:**

Hospitalized patients experience sleep disruption with consequential physiological and psychological effects. Surgical patients are particularly at risk due to surgical stress and postoperative pain. This systematic review aimed to identify non-pharmacological interventions for improving sleep and exploring their effects on sleep-related and clinical outcomes.

**Methods:**

A systematic literature search was performed in accordance with PRISMA guidelines and was preregistered on the Open Science Framework (doi: 10.17605/OSF.IO/EA6BN) and last updated in November 2023. Studies that evaluated non-pharmacological interventions for hospitalized, adult patients were included. Thematic content analysis was performed to identify hypothesized mechanisms of action and modes of administration, in collaboration with a patient partner. Risk of bias assessment was performed using the Cochrane Risk Of Bias (ROB) or Risk Of Bias In Non-Randomized Studies – of Interventions (ROBINS-I) tools.

**Results:**

A total of 59 eligible studies and data from 14 035 patients were included; 28 (47.5%) were randomized trials and 26 included surgical patients (10 trials). Thirteen unique non-pharmacological interventions were identified, 17 sleep measures and 7 linked health-related outcomes. Thematic analysis revealed two major themes for improving sleep in hospital inpatients: enhancing the sleep environment and utilizing relaxation and mindfulness techniques. Two methods of administration, self-administered and carer-administered, were identified. Environmental interventions, such as physical aids, and relaxation interventions, including aromatherapy, showed benefits to sleep measures. There was a lack of standardized sleep measurement and an overall moderate to high risk of bias across all studies.

**Conclusions:**

This systematic review has identified several sleep interventions that are likely to benefit adult surgical patients, but there remains a lack of high-quality evidence to support their routine implementation.

## Teaser

Hospitalized patients experience sleep disruption with consequential physiological and psychological effects. This systematic review has identified several sleep interventions, such as eye masks and ear plugs, that are likely to benefit adult surgical patients, but there remains a lack of high-quality evidence to support their routine implementation.

## Introduction

Sleep is vital for recovery from injury^1^. As well as the removal of metabolic waste, sleep is important for cellular responses in the body^2,3^. Sleep deprivation, therefore, can have significant adverse effects on normal physiological processes, including increased susceptibility to infection, overactivation of the sympathetic nervous system and increased risk of delirium^4–6^. Despite these risks, sleep deprivation is common in hospital, and particularly in the perioperative setting where environmental disturbances are common, pain and anxiety can affect sleep quantity and quality, and wards can be high-turnover and manage acute conditions and complications^7^.

Patients undergoing elective surgery experience a significant insult during major surgery and may take months to return to their functional and physiological baseline, if at all. During this time they are at risk of surgical complications that may delay their recovery and apply further systemic stress^8^. Enhanced Recovery After Surgery (ERAS) guidelines for postoperative care have been widely implemented across the world to improve mobility, diet, fluid status and analgesia, and reduce unnecessary interventions (for example routine nasogastric placement after colorectal resection)^9^. However, no ERAS guidelines currently include sleep quantity or quality. Given the negative effects of sleep deprivation, interventions to improve sleep after surgery have the potential to both moderate the surgical stress responses and mediate high compliance with other components of the ERAS pathway (for example by improving appetite and energy for mobilization).

Although other systematic reviews evaluating the effectiveness of sleep interventions for hospital inpatients exist, these have largely focused on critical care populations and drug therapies^10,11^. Pharmacological therapies typically have unattractive side-effect profiles that may hinder postoperative recovery^12^. Further research in non-pharmacological interventions (NPIs) for use in non-critical care areas including surgical wards is urgently needed^13^. In addition, traditional methods for sleep measurement such as highly controlled sleep studies are not feasible in a hospital environment. Future trials of NPIs in the surgical setting will have to adopt innovative, but validated methods for sleep measurement. The objectives of this systematic review were therefore three-fold: first, to identify and evaluate interventions tested out to improve sleep quality and quantity; second, to identify the approaches to measuring sleep in hospitalized adult patients; and third, to extract other sleep-associated health-related outcomes. The overall aim was to inform the co-development of a future randomized trial in patients undergoing surgery.

## Method

### Study design and search strategy

A systematic review was conducted in accordance with the Preferred Reporting Items for Systematic Reviews and Meta-analysis (PRISMA) guidelines and the protocol was preregistered on the Open Science Framework (doi: 10.17605/OSF.IO/EA6BN)^14^. The search included papers published up until 27 December 2020 and was developed through iterative preliminary searches using PubMed (*Supplementary methods*). The search was last updated in November 2023. Where available, search strategies from existing systematic reviews in similar topic areas were used to further inform the search strategy. In addition to this, the Cochrane Database of Systematic Reviews (CDSR) was reviewed to identify reviews of sleep NPIs, and a hand search was further carried out to ensure all studies that met the inclusion criteria were identified. As the aim of the review was to provide a thorough overview of a topic area, a search of the grey literature was also conducted using System for Information on Grey Literature in Europe (SIGLE). The following sources were searched without date restrictions: PubMed and Ovid via Medline. Endnote (Clarivate Analytics) was used to collate all references from the databases and identify duplicate studies. Reasons for exclusion of any full-text articles were recorded.

### Study inclusion and exclusion criteria

Inclusion and exclusion criteria were based upon study, patient and hospital characteristics. Primary research studies including RCTs, prospective or retrospective observational studies were eligible. Studies reporting NPIs in adult, non-ventilated patients without pre-existing sleep disorders were included. Any studies with pharmacological interventions only or mixed interventions which could not be disaggregated or with mixed adult and paediatric data or ventilated and non-ventilated patients which could not be disaggregated were excluded.

### Patient and public involvement

The idea for this review arose from discussion with surgical patients and was highlighted during a perioperative patient advisory group meeting with Patients and Research Together (PART) from Bowel Research UK. As this review targeted a high-priority area for patients, the study protocol and process was co-produced in partnership with a patient advocate (S.B.). The patient representative formed part of the core study steering group and was invited to participate in all aspects of the review. This included designing the search strategy, performing the thematic analysis, drafting and reviewing the study manuscript, and drafting the visual abstract. The final manuscript was sent back to members of the PART group for comment. In order to report the impact of patient and public involvement activity within this review, the Guidance for Reporting Involvement of Patients and the Public (GRIPP2) short-form reporting checklist was used^15^ (*Table S1*).

### Data analysis

Data extraction was performed by two independent reviewers (R.A. and B.H.). Any discrepancies were resolved during a study group meeting including the senior author (J.G.), until a consensus was achieved. Study characteristics, including study design, sample size and country of origin were presented. The reporting transparency was assessed by whether authors had cited the corresponding Enhancing the QUAlity and Transparency Of health Research (EQUATOR) network guideline^16^. Participant characteristics such as hospital setting, age groups, disease types and use of sedating analgesics were presented. For studies including surgical patients, included operation types were also described.

The data analysis plan was co-developed with a patient partner and structured around the three predefined research objectives. First, NPIs were extracted verbatim and then combined to reduce redundancy to form unique NPI definitions. A three-stage process of thematic analysis was undertaken based on the hypothesized mechanism of action and method of administration. Conceptual themes were extracted and underwent double coding. The themes were reviewed and refined in a patient advisory group meeting (R.A., J.G., S.B.). The final thematic groups were reviewed across the study steering group before being accepted. The frequency of reporting of each NPI (and thematic group) across different patient groups and hospital settings was explored to identify differences in their patterns of application. Next, using evidence synthesis and critical appraisal the directionality of effect of NPIs (and thematic groups) on hospitalized adult patients was described to identify early signals of patient benefit. Due to predicted heterogeneity of study populations and interventions, meta-analysis was not preplanned. Second, measures of sleep that were used in included studies were extracted and grouped into self-reported or physiological measures. Differences in timing, frequency and types of measure used across patient groups and hospital settings were explored and compared. Third, any short-term health-related outcomes relevant to sleep were extracted. To enrich this process, the Cochrane Risk of Bias (ROB) tool was used to assess for risk of bias in randomized studies, and the risk of bias in non-randomized studies of intervention (ROBINS-1) tool was used for non-randomized studies.

## Results

**Figure 1:**
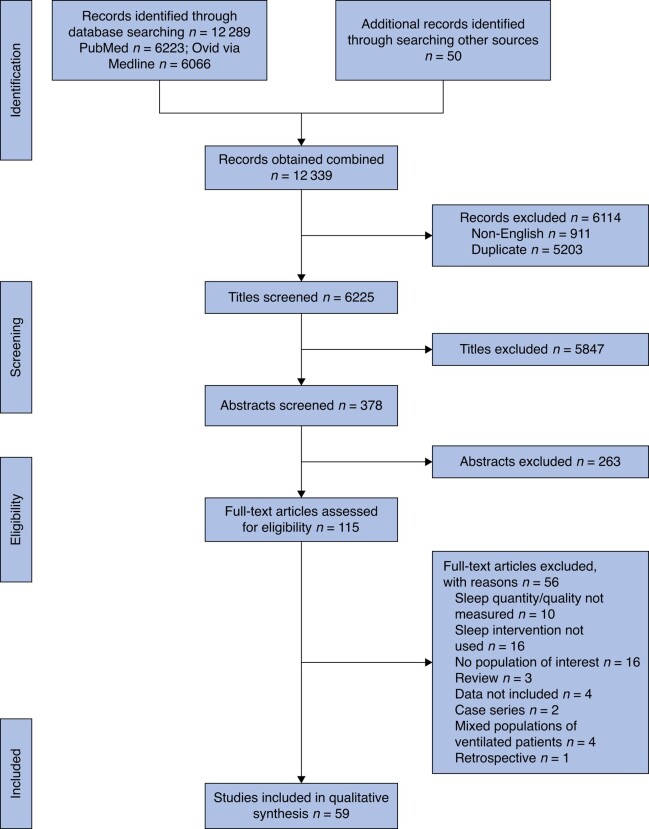
PRISMA flow chart

A total of 59 full-text studies were included in data extraction and evidence synthesis (*Fig. 1*)^17–75^. *Table S2* displays study and patient characteristics. Of 59 eligible studies, 28 studies were RCTs, ten were non-randomized interventional studies and 21 were prospective cohort studies. In total, data from 14 035 participants was included in this review. The reporting transparency of included studies was poor; only three studies reported the use of EQUATOR network guidelines^21,50,60^ and one study used the Transparent Reporting of Evaluations with Nonrandomized Designs (TREND) reporting guidelines for non-randomized clinical trials^73^. No conflicts of interest or funding discrepancies were identified.

Of the 59 included studies of sleep NPIs, 17 (28.8%) were conducted in a type of critical care unit^19,22,29,37,42,45,51–54,57,58,61,65,69,72,74^. Other common hospital wards were medical specialty wards (*n* = 20), mixed medical-surgical units (*n* = 8) and oncology wards (*n* = 5). Twenty-six (44.1%) studies included surgical patients, with only ten studies conducted on a surgical ward. This included patients in surgical critical care units, and recovering from abdominal surgery, burns, cardiothoracic and neurosurgery. The most common surgical specialty ward was cardiothoracic surgery. Of all surgical studies, only one study reported concurrent use of an enhanced recovery pathway^47^.

### Thematic coding and analysis results

After iterative coding and refinement, two major themes were identified relating to the hypothesized mechanism of action: improving the sleep environment, and relaxation and mindfulness. Two further themes arose related to method of administration: self-administered and carer-administered. The two most common NPIs were environmental modifications to the patient’s environment and light therapy. Apart from one study, all studies evaluating relaxation NPIs were clinician administered. In surgical patients, the most common theme was environmental, specifically multimodal interventions and physical aids. Key feedback points from involvement of patients in iterative coding and interpretation were two-fold: that future trials in this area must be co-produced to ensure that they are feasible, acceptable and speak to patients’ true lived experience; that sleep disturbance in hospital is likely to be multifactorial and individual interventions are unlikely to succeed in isolation.

### Environmental NPIs

Environmental NPIs focused on minimizing sleep disturbances created from the patient’s environment, as further described in *Table 1*. Sleep-promotion aids were incorporated within intervention bundles, with ten studies investigating the direct effects of ear plugs and eye masks^19,37,40,47,61,66,69,72,74,75^. Nine of these studies reported statistically significant improvements in sleep domains^19,37,40,47,61,66,69,72,74^. Twenty (33.9%) studies focused on improving the sleep environment for hospitalized patients, including creating quiet time protocols for the patients and caring staff. The results of these studies were inconsistent across all studies. Improvements were reported in the duration of sleep, sleep efficiency and subjective ratings of sleep quality (*Table 2*). Results from nurse-led observations showed improvements in the number of patients asleep during the intervention. As the protocols assessed across the studies involved several different interventions, a definitive cause-and-effect relationship cannot be established. Seven (12.5%)^21,23,24,34,36,50,71^ studies investigated bright light exposure, and were mainly conducted on geriatric or psychiatric patients. Reported improvements following bright light exposure were in sleep duration and sleep quality. One study exploring the effects of a privacy curtain designed to increase speech privacy and reduce noise disturbances reported an increase in sleep measure score and an increase in the patient’s self-reported ability to rest^43^.

**Table 1:** Description of environmental and relaxation non-pharmacological interventions (NPIs)

Theme	NPIs
Environmental	Physical aids (for example ear plugs, eye masks)
	Multimodal environmental changes (for example turning ward lights off, reducing visitor times)
	Light therapy
Relaxation/mindfulness	Aromatherapy
	Acupuncture
	Guided imagery
	Relaxation therapy
	Relaxation and imagery combined
	Music therapy
	Milk-honey mixture
	Relaxation/mindfulness protocol
Both	Psychological counselling and physical sleep-promoting aids

**Table 2:** Summary of non-pharmacological interventions (NPIs) for adult hospital inpatients

Article (first author and year)	Type of NPI	Description of NPI	Sleep-related outcomes	Direction of effect	Outcomes
Aksu, 2018^17^	Relaxation/mindfulness	Relaxation therapy	Sleep quality, sleepiness	Positive	Participants in the intervention group showed significantly greater improvements than the control group in sleep-related outcomes (*P* < 0.05)
Alparslan, 2016^18^	Relaxation/mindfulness	Relaxation therapy	Sleep quality and sleep latency	Positive	Participants in the intervention group who received progressive muscle relaxation training showed significantly greater improvements in sleep quality
Bani Younis, 2019^19^	Environmental	Physical aids	Sleep quality, sleep quantity, number of awakenings, sleep latency, depth of sleep	Positive	Participants in the intervention group slept more hours and reported significantly better sleep quality compared with participants in the control group, following the use of eye masks and earplugs
Bartick, 2010^20^	Environmental	Multimodal environmental changes	Sleep quantity, sleep quality, sleep latency, changes in the use of sleep medication	None	No improvements were seen in any sleep-related measures
Canazei, 2019^21^	Environmental	Light therapy	Sleep quantity, sleep quality, sleep latency, sleep efficiency and changes in the use of sleep medication	Positive	Daytime sleepiness was significantly improved in the group receiving bright light therapy compared with the control group (*P* = 0.004). The light intervention group also had improvements in overall sleep quality (*P* = 0.034), reduced sleep latency (*P* = 0.029) and sleep disturbances (*P* = 0.036), and increased sleep duration (*P* = 0.026)
Cho, 2013^22^	Relaxation/mindfulness	Aromatherapy	Sleep quality, sleep quantity and sleep latency	Positive	Participants in the aromatherapy group showed improvements in sleep quality (*P* = 0.001) compared with conventional nursing interventions during their stay in ICU
Chong, 2013^23^	Environmental	Light therapy	Sleep quantity, number of awakenings,	None	No statistically significant difference in sleep parameters
De Rui, 2015^24^	Environmental	Light therapy	Sleep quantity, sleep quality, sleep latency, sleep efficiency, sleepiness	None	Treatment with bright light therapy did not show beneficial effects on sleep-related outcomes
Dobing, 2017^25^	Environmental	Multimodal environmental changes	Sleep quality, changes in the use of sleep medication	None	No significant differences were found in sleep duration or sleep quality
Ducloux, 2013^26^	Relaxation/mindfulness	Relaxation therapy	Sleep quality	None	There were no significant improvements in sleep quality for patients receiving relaxation therapy
Fakhr-Movahedi, 2018^27^	Relaxation/mindfulness	Milk-honey mixture	Sleep quality and sleep latency	Positive	On the third day of admission, there was a significant difference in sleep scores between the intervention and control group (*P* = 0.001)
Fan-Lun, 2019^28^	Environmental	Multimodal environmental changes	Sleep quality and changes in the use of sleep medication	None	No improvements were seen in self-reported sleep quality
Faraklas, 2013^29^	Environmental	Multimodal environmental changes	Sleep quality, sleep efficiency, and changes in the use of sleep medication	Positive	Participants postintervention saw a significant improvement in falling asleep quickly (*P* = 0.022)
Farrehi, 2016^30^	Environmental	Multimodal environmental changes	Sleep quantity and sleepiness and changes in the use of sleep medication	None	No statistically significant improvements in sleep between control and intervention group
Garcia, 2018^31^	Relaxation/mindfulness	Acupuncture	Fatigue	Positive	Significant improvements were found in mean(s.d.) scores in drowsiness (−0.6(1.8); *n* = 57; *P* = 0.020) and fatigue (−0.4(1.1); *n* = 67; *P* = 0.008) following acupuncture, compared with baseline
Gardner, 2009^32^	Environmental	Multimodal environmental changes	Number of participants asleep, sleepiness	Positive	Greater number of participants were asleep in the intervention group compared with the control group (*P* < 0.01)
Gathecha, 2016^33^	Environmental	Multimodal environmental changes	Sleep quantity, sleep efficiency	Positive	Total sleep time, computed from sleep diaries, demonstrated significant overall mean difference of 49.6 min (standard error (s.e.) = 21.1, *P* < 0.05)
Gimenez, 2017^34^	Environmental	Light therapy	Sleep quantity, sleepiness	Positive	Actigraphic sleep duration improved by 5.9 min (95% c.i. 0.6 to 11.2; *P* = 0.03) per hospitalization day with interventional lighting instead of standard lighting. After 5 days of hospitalization, sleep duration in the lighting intervention rooms increased by 29 min, or a relative 7.3% compared with standardly lit rooms
Hajibagheri, 2014^35^	Relaxation/mindfulness	Aromatherapy	Sleep quality, sleep quantity, sleep latency, sleep efficiency and changes in the use of sleep medication	Positive	Sleep latency, sleep duration and sleep efficiency scores improved after the intervention and the total sleep quality score decreased after the intervention (*P* < 0.05), indicating an improvement in sleep-related outcomes
Henriksen, 2020^36^	Environmental	Light therapy	Sleep quantity, sleep fragmentation index, sleep efficiency, number of wake episodes	Positive	Sleep efficiency was significantly higher amongst participants in the intervention group compared with the placebo group (92.6% *versus* 83.1%, *P* = 0.027). There were fewer nights of interrupted sleep in the intervention group (29.6%) *versus* in the placebo group (43.8%)
Jones, 2012^37^	Environmental	Physical aids	Sleep quality, sleep quantity	None	Eye masks and earplugs did not improve the participants’ quality of sleep
Kuon, 2019^38^	Relaxation/mindfulness	Massage	Fatigue	Positive	Based on subjective reporting of sleep quality, 73% of participants receiving the massage therapy reported ‘better’ or ‘much better’ sleep the following night after intervention
Lareau, 2008^39^	Environmental	Multimodal environmental changes	Sleep quality, sleep quantity, sleep efficiency and changes in the use of sleep medication	None	There was no statistically significant difference in sleep quality or duration between the intervention and control group
Le Guen, 2014^40^	Environmental	Physical aids	Sleep quality, sleep quantity, sleepiness, sleep latency and sleep efficiency	Positive	In the intervention group receiving ear plugs and eye masks, participants had fewer sleep disruptions and the need for adjunctive rest above 15 min was less frequent (50%, 95% c.i. 20 to 80 *versus* 95% c.i. 85 to 100, *P* = 0.001)
Lee, 2017^41^	Environmental	Multimodal environmental changes	Sleep quality, sleep quantity	Positive	Participants in the intervention group reported significantly (*P* = 0.015) lower mean(s.d.) sleep disturbance scores (53.1(14.5)) compared with the control group (71.9(18.8))
Leong, 2021^75^	Environmental	Physical aids	Sleep quality, sleep depth and sleep latency	Negative	The use of eye masks and ear plugs on postoperative days 1–3 did not improve quality of sleep after major abdominal surgery
Li, 2011^42^	Environmental	Multimodal environmental changes	Sleep quality, sleep efficiency, sleep latency, sleepiness	Positive	The intervention group reported better sleep quality (t = −2.28, *P* = 0.027) and sleep efficiency (t = −2.03, *P* = 0.047) compared with the control group
Locke, 2017^43^	Environmental	Privacy curtain	Ability to rest, overall improvement in sleep measures	Positive	Patients on the refurbished nursing unit and rooms with the privacy curtain rated their ability to rest at night higher than average compared with patients on the standard nursing unit and standard privacy curtain
Lytle, 2014^44^	Relaxation/mindfulness	Aromatherapy	Sleep quality, depth of sleep, sleep latency and number of awakenings	None	There were no statistically significant improvements in sleep measures in the intervention group
Maidl, 2014^45^	Environmental	Multimodal environmental changes	Depth of sleep, sleep latency, number of awakenings, sleep efficiency and sleep quality	None	There was no statistically significant effect of the intervention on sleep measures
McDowell, 1998^46^	Relaxation/mindfulness	Non-pharmacological sleep protocol	Sleep quality and changes in the use of sleep medication	Positive	The sleep protocol had a strong association with quality of sleep amongst patients who had never received a sedative for sleep. Good sleep was reported in 51% of patient-days when all three parts of the protocol were received. When none of the protocol was received, poor sleep was reported in 45% of patient-days. More patients reported significantly improved quality of sleep (χ^2^ = 71.9, *P* < 0.001) when more parts of the protocol were received
Menger, 2018^47^	Environmental	Physical aids	Sleep quality, sleep latency	Positive	Menger *et al.* assessed quality of sleep using a scale from 1 (excellent) to 5 (very poor) and patients in the intervention group reported a better quality of sleep (median, i.q.r. (range): 3, 2–4 (1–5) *versus* 4, 3–5 (1–5), *P* = 0.05)
Nooner, 2016^48^	Relaxation/mindfulness	Relaxation and imagery combined	Fatigue and sleep disturbances	Positive	Results showed a trend towards improvement in sleep quality, with reduced sleep disturbance and more refreshing sleep, amongst participants receiving guided imagery
Norton, 2015^49^	Environmental	Multimodal environmental changes	Sleep quality	Positive	Overall sleep rating was significantly improved, from 47% (352 of 749) reporting sleep as good or excellent at baseline to 69% (540 of 783) at follow-up (*P* < 0.001)
Obanor, 2021^74^	Environmental	Physical aids	Sleep quantity, sleep quality, sleep depth and sleep latency	Positive	The average RCSQ score, used to measure sleep-related outcomes, in the intervention group was significantly higher at 64.5 (95% c.i. 58.3 to 70.7, *P* = 0.0007), compared with the control group with an average RCSQ score of 47.3 (95% c.i. 40.8 to 53.8)
Okkels, 2020^50^	Environmental	Light therapy	Sleep quality, sleep latency, sleep efficiency, sleepiness and changes in the use of sleep medication	None	Non-significant changes were reported in sleep quality in participants in the intervention group
Olson, 2001^51^	Environmental	Multimodal environmental changes	Number of participants asleep	Positive	The percentage of patients observed to be asleep was significantly higher during the implementation of the ‘quiet time’ protocol compared with the control period before the intervention started. Patients observed during the intervention period were 1.6 times more likely to be asleep during the designated ‘quiet time’ compared with the control period (*P* < 0.001)
Ong, 2020^52^	Relaxation/mindfulness	Guided imagery or virtual reality	Sleep quality	None	No statistically significant difference was observed when assessing sleep quality between the intervention and control group
Ozlu, 2017^53^	Relaxation/mindfulness	Aromatherapy	Sleep quality, sleep latency	Positive	The mean(s.d.) RCSQ score, used to measure sleep-related outcomes, was 53.80(13.20) in the experimental group and 20.08(9.71) in the control group, a difference that was statistically significant (*P* < 0.001)
Patel, 2014^54^	Environmental	Multimodal environmental changes	Sleep efficiency	Positive	The bundle of interventions led to an increased mean(s.d.) sleep efficiency index (60.8(3.5) before *versus* 75.9(2.2) after, *P* = 0.031)
Pati, 2016^55^	Relaxation/mindfulness	Guided imagery or virtual reality	Sleep quality and change in the use of sleep medication	None	There was no statistically significant difference in sleep quality between the experimental and control group
Pattison, 1996^56^	Environmental	Multimodal environmental changes	Sleep efficiency, number of awakenings	None	There was no statistically significant difference in sleep improvement between the control and intervention ward
Richards, 1998^57^	Relaxation/mindfulness	Massage	Sleep quantity, number of awakenings, changes in the use of sleep medication and sleep latency	Positive	Total sleep time for the group of participants receiving the back massage was 62.5 min longer and latency to sleep onset was 6.8 min less than those values in the control group. Sleep efficiency index was 14.7% higher in the massage group than in the control group. The back-massage group spent 35.0 min in REM sleep, which was longer than the 25 min for REM sleep in the control group
Richardson, 2003^58^	Relaxation/mindfulness	Relaxation and imagery combined	Sleep quantity, sleep latency, depth of sleep and number of awakenings	None	The overall effect of the intervention on sleep scores was not significant
Ryu, 2012^59^	Relaxation/mindfulness	Music	Sleep quality, and sleep quantity	Positive	Participants in the intervention group reported that sleep quantity and duration were significantly higher than in the control group (t = 3.181, *P* = 0.002, t = 5.269, *P* < 0.001 respectively)
Scarpa, 2017^60^	Both	Both	Sleep quality, sleep quantity, sleep latency, changes in the use of sleep medication	Positive	Psychological counselling reduced the postoperative impairment of sleep quality (odds ratio 0.27, 0.10 to 0.73)
Scotto, 2009^61^	Environmental	Physical aids	Sleep quality, sleep quantity and sleep efficiency	Positive	Total sleep satisfaction scores were significantly better for the intervention group (*P* = 0.002)
Silvius-Byron, 2014^62^	Environmental	Multimodal environmental changes	Sleep quality, and ability to rest	None	The restriction of visitors and designated rest period did not improve the patients’ perception of rest or how long it took them to go to sleep
Smith, 2002^63^	Relaxation/mindfulness	Massage	Sleep quality and sleep latency	None	No improvement in subjective sleep quality was shown for patients in the treatment massage group
Spence, 2011^64^	Environmental	Multimodal environmental changes	Sleep quality, use of sleep promoting aids	None	Sleep quality and quantity were assessed through the number of noise events, which reduced sleep. Relaxation and sleep promotion aids did not reduce the number of events per participant
Su, 2013^65^	Relaxation/mindfulness	Music	Sleep quality and quantity	Positive	Participants receiving the intervention had improved self-reported sleep quality compared with those in the control group
Sweity, 2019^66^	Environmental	Physical aids	Sleep quality, changes in the use of sleep medication	Positive	The mean sleep quality score was 6.33 (95% c.i. 5.89 to 6.77) in the intervention group, compared with 5.09 (95% c.i. 4.66 to 5.52) in the control group (*P* < 0.001)
Tas, 2014^67^	Relaxation/mindfulness	Acupuncture	Sleep quality, sleep latency	Positive	Tas *et al.* demonstrated a statistically significant decrease (*P* < 0.001) in insomnia after the acupuncture treatment compared with baseline
Thomas, 2012^68^	Environmental	Multimodal environmental changes	Sleep quality, sleep latency	Positive	There was no statistically significant improvement in total sleep time or number of awakenings. However, there was a significant improvement in sleep latency during phase 2 of the study
Van Den Ende, 2022^73^	Both	Both	Sleep quantity, sleep quality, sleep latency and sleep efficiency	Positive	Implementation on non-pharmacological interventions demonstrated a 40- to 45-min increase in sleep quantity. Patients in the control group had a median sleep time of 6 and 5 min and patients in the intervention group had a median sleep time of 6 and 45 min (*P* < 0.001)
Van Rompaey, 2012^69^	Environmental	Physical aids	Sleep quality	Positive	Sleep perception was assessed using a non-validated sleep quality questionnaire containing five dichotomous questions. Patients with the earplugs demonstrated significantly better sleep after the first night (*P* = 0.042)
Vitinius, 2014^70^	Relaxation/mindfulness	Aromatherapy	Sleep quality, sleep quantity, dream quality	None	Application of the odorant showed no significant differences in sleep quality between the placebo and intervention group
Wakamura, 2001^71^	Environmental	Light therapy	Sleep quantity, melatonin secretion	Positive	Melatonin secretion was measured and showed an increase in three (75%) patients during bright light exposure. Bright light exposure prolonged ‘Time in Bed’ (*P* < 0.05), increased ‘immobile minutes’ (*P* < 0.05), and delayed ‘Get up Time’ (*P* < 0.01)
Yazdannik, 2014^72^	Environmental	Physical aids	Sleep quality, sleep efficiency	Positive	There were significant differences (*P* < 0.001) in sleep effectiveness between the treatment night and control night

RCSQ, Richards–Campbell Sleep Questionnaire; REM, rapid eye movement.

### Relaxation NPIs

Of the 21 studies investigating relaxation NPIs, 13 (61.9% of all relaxation NPI studies) showed statistically significant improvements in sleep-related outcomes. Acupuncture was evaluated in two studies, and both showed statistically significant improvements in sleep outcome. Garcia *et al.* reported a significant improvement in drowsiness and fatigue compared with baseline for patients and Tas *et al.* demonstrated a statistically significant decrease (*P* < 0.001) in insomnia amongst patients receiving acupuncture treatment^31,67^. Massage therapy showed improvements in the quantity of sleep in only one study, whereby the total sleep time for participants receiving the back massage group was 62.5 min longer than in the control group, as well as a shorter latency to sleep onset^57^. Progressive muscle relaxation therapy showed significant improvements in two studies where participants in the intervention group had greater improvements in sleep-related outcomes (*P* < 0.050) compared with the control group^17,18^. Relaxation therapy combined with guided imagery was found to reduce fatigue and sleep disturbances amongst participants^48^. The effects of aromatherapy on sleep were variable; however, three studies reported improvements in sleep quality and total sleep scores^22,35,53^. One study exploring the benefits of a milk-honey mixture reported significant improvements in sleep scores between the intervention and control group^27^. A study involving back rubs, warm drinks and relaxation tapes reported positive dose-wise effects on chart-abstracted and self-reported sleep^46^.

### Sleep measurement

The outcomes relating to sleep quantity and quality varied significantly across all studies, with the majority involving subjective measurements (*Table 3*). Twenty-four (40.1%) studies reported sleep duration or total sleep time (TST), 43 (72.9%) studies reported sleep quality and 24 (40.7%) studies reported sleep latency. Most studies used self-reported measures of sleep: 53 studies administered questionnaires to the participants and four studies reported sleep logs. Five studies employed the use of a questionnaire completed by the caring staff or designated surveyor. The three most common types of validated questionnaire employed by studies included: the Richards-Campbell Sleep Questionnaire (RCSQ), the Pittsburgh Sleep Quality Index (PSQI) and the Verran and Snyder-Halpern (VSH) sleep scale. Five (8.9%) studies created a study-specific questionnaire, with no pilot testing or validation^47,56,59,68,69^. Objective measurements were only reported in eight (13.6%) studies: six used actigraphy^33,34,36,40,71,73^ and two used polysomnography^57,65^. Actigraphy measurements were recorded using an actigraphy wristwatch.

**Table 3:** Measures of sleep quantity and quality

Sleep-related outcomes	Method(s) of measurement
Sleep quality	Self-reported questionnaire^17–22,25,27–30,35,37,39,40,42,44–46,49,50,52–55,59–67,69,72–75^, sleep log^18,26,33,41^, patient interviews^18,46,49,53^, nurse-led observations^40,47^, polysomnography^65^
Sleep latency	Self-reported questionnaire^18–22,24,27,35,40,42,44,45,47,50,53,58,60,63,67,68,74,75^, polysomnography^57,65^, actigraphy^40,73^
Sleep efficiency	Self-reported questionnaire^17,21,24,29,33,35,36,39,40,42,45,50,52–54,56,57,60,61,68,72^, actigraphy^40,73^
Sleepiness	Self-reported questionnaire^17,24,30,32,34,40,42,50^
Ability to rest	Self-reported questionnaire^40,43,62^
Fatigue	Self-reported questionnaire^30,31,38,48^
Satisfaction of sleep	Self-reported questionnaire^26^
Dream quality	Self-reported questionnaire^70^
Use of sleep-promoting aids	Self-reported questionnaire^64^
Number of participants asleep	Nurse-led observations^32,51^
Number of awakenings	Nurse-led observations^23,36^, actigraphy^36^, polysomnography^57^, self-reported questionnaire^19,44,45,56,58^
Sleep fragmentation index	Actigraphy^36^
Total sleep time or sleep quantity	Actigraphy^33,34,36,40,71,73^, polysomnography^57,65^, nurse-led observations^19,20,23,36,39,51^, self-reported questionnaire^20–22,37,40,41,58,59,61,74^
Time in each sleep stage	Polysomnography^57,65^
Depth of sleep	Polysomnography^57,65^, self-reported questionnaire^19,44,45,58,74,75^
Melatonin secretion	Salivary samples^71^
Changes in the use of sleep medication	Self-reported questionnaire^17,20,21,25,28–30,35,39,46,50,55,57,60^, nurse-led observation/medical records^25,46,66^

### Reporting of clinical outcomes

The reporting of clinical outcomes was inconsistent across studies, with the most common physiological outcomes including vital signs (*n* = 7), depression (*n* = 11), delirium (*n* = 7), nausea (*n* = 4), pain (*n* = 14), anxiety (*n* = 14) and duration of hospitalization (*n* = 18). Studies conducted on surgical patients also reported changes to postoperative pain and duration of hospital stay. No studies reported the effect of sleep on appetite, mobility, infection or wound healing. The majority of NPIs showing an improvement in clinical outcomes (typically anxiety and delirium) were themed as relaxation/mindfulness.

### Risk of bias assessment

In the ROB-2 assessment, most RCTs performed adequate randomization processes, commonly through a computer randomization method and drawing a random number. However, a few studies lacked specific details of the method of sequence generation. Allocation concealment was seldom reported across RCTs. Due to the nature of NPIs, most studies failed to blind participants or personnel involved. Therefore, all studies were at risk of performance and detection bias. Considerable risk of bias was present regarding the measurement of outcomes due to the use of self-reported questionnaires. In the ROBINS-I assessment, serious risk of bias was present due to the measurement of outcomes as well as the use of subjective sleep measures, which are at a high risk of performance bias. Several studies reporting dropouts or losses to follow-up were at risk of attrition bias, particularly as insufficient detail regarding the reasons for missing outcome data were documented. Most non-randomized studies failed to conduct effect analyses on all the reported outcomes, introducing a risk of selective reporting based on results.

## Discussion

This review classified NPIs to improve sleep for hospitalized patients according to the mechanism of action and mode of administration, working closely with patient partners. The study identified signals of benefit in both environmental and mindfulness and relaxation NPIs. Environmental modifications, physical sleep adjuvants and aromatherapy were most likely to improve sleep duration and quality amongst surgical patients. Included studies typically had moderate or high risk of bias, limiting the overall certainty in recommendations. For example, objective measures of sleep (for example actigraphy) alongside patient-reported measures are recommended to accurately evaluate the effectiveness of NPIs in the future. High-quality randomized trials are now needed to strengthen the evidence base and inform the introduction of sleep interventions to ERAS protocols for surgical patients. The patient partners in this mixed methods study highlighted that the development of NPIs should be performed with patients as equal partners to ensure acceptability, feasibility and relevance to surgical populations.

Poor sleep quality and sleep disruptions are common for hospitalized patients^76^. The patient advisory group highlighted sleep as one of the most disturbing influences on wellbeing during postoperative recovery. Among factors such as pain and medication effects which are common in the postoperative setting, interruptions caused by noise and light levels have been shown to contribute towards disrupted sleep^77^. Given the physical and cognitive limitations of hospitalized patients, most environmental interventions in the review were passive in nature and were administered to, rather than by, patients. Interventions designed to minimize environmental noise were multifactorial and included: clustering care activities, ensuring designated quiet time at night, dimming lights and closing doors if necessary. Currently, the use of NPIs during postoperative recovery is not standardized practice across all hospitals. Consideration of both self-administered and clinical-administered NPIs will be essential in maintaining an optimum and adaptable sleep environment for patients in the future. Optimization of the hospital environment is attainable with multidisciplinary support. However, due to the multifactorial nature of sleep hygiene protocols, the review is unable to ascertain the specific components that benefitted sleep the most.

Implementation of multifactorial environmental NPIs was variable and clinical activities, sometimes necessarily (for example to maintain safety), took precedence over the NPI. It is important that caring staff are motivated to reduce the most disruptive factors to patients’ sleep. Therefore, future improvement initiatives should be co-designed with deep stakeholder engagement to both ensure that proposed NPIs are feasible and acceptable, and to increase awareness of the importance of sleep hygiene^78^. This should follow National Institute for Health and Care Research (NIHR) and Medical Research Council (MRC) complex intervention development recommendations^79^. Bright light therapy demonstrated improvements in sleep duration and efficiency. However, these studies were largely conducted amongst geriatric and psychiatric patients, which highlights a need for further research amongst surgical patients.

The use of ear plugs and eye masks to minimize sleep disruption proved to be a plausible and practical NPI across medical and surgical patients^19,37,40,47,61,66,69,72,74^. The low quality of studies and moderate to serious risk of bias is in accordance with other reviews^10,80^. Compliance with the use of physical sleeping aids was also variable. Relaxation/mindfulness interventions aim to induce anti-anxiolytic effects and restore the resting state. Aromatherapy oils resulted in statistically significant improvements in patients’ self-reporting quality of sleep, consistent with prior reviews on the effect of aromatherapy. Acupuncture had consistent positive findings on subjective measures of sleep. However, the small sample sizes and poor compliance with self-reported questionnaires affected the validity of the results.

Identifying the mode of administration of sleep intervention is crucial for optimizing effectiveness and enhancing patient comfort. Eye masks and ear plugs showed benefits across sleep domains, and both are self-administered, allowing patients to be part of their care and providing an individualized method for improving sleep. Relaxation therapies including aromatherapy and massage therapy require the involvement of additional specialized individuals to deliver the intervention. This ought to be taken into account when assessing the feasibility of implementing a relaxation/mindfulness intervention in clinical practice.

Currently, a diverse range of methods of measuring sleep-related domains exist, with many studies using an unvalidated questionnaire. The use of unvalidated questionnaires can reduce the credibility of the data and outcomes may be subject to measurement error. Self-reported questionnaires had poor compliance and patient symptoms appeared to hinder the collection of self-reported measurements of sleep quality and quantity—a vital consideration for future trials. No studies evaluated the effect of sleep interventions on validated patient-reported measures of patient recovery from surgery such as Quality of Recovery-15 (QoR-15)^81^. Objective measures of sleep were seldom used, which may largely be due to the extensive technology required to record and analyse the recordings, rendering methods such as polysomnography expensive and labour-intensive^82^. Novel wearable technologies for sleep measurement pose an attractive, accessible target for objective sleep monitoring^83^.

The aetiology of sleep disruption in surgical patients is multifactorial. This review identified several NPIs that demonstrated significant improvements in sleep and related clinical measures. Patients undergoing surgery may benefit from both environmental NPIs such as sleep masks and ear plugs and relaxation/mindfulness NPIs, such as muscle relaxation therapy or aromatherapy. These are unlikely to interfere with patient treatment or affect the safety of other patients, but may significantly reduce anxiety, stress and sleep disturbance during the perioperative period^84^. The cost of implementing sleep-improving interventions is likely to vary significantly. Healthcare providers must ensure the sustainability and accessibility of sleep-improving interventions prior to implementation. Eye masks and ear plugs are self-administered interventions and require minimal input from additional specialists, in contrast to relaxation/mindfulness interventions, which may be associated with higher costs; it was not possible to compare the clinical or cost effectiveness of the included NPIs here. The findings from this study should guide the collaborative development of future RCTs, focusing on improving sleep quality and quantity in surgical wards, with active involvement and input from patients.

This review had several limitations. First, the aim of this review was not to estimate the exact efficacy of all sleep NPIs by assimilating the available evidence. The extensive scope of the subject area and heterogeneity between study interventions and outcomes meant meta-analysis was not possible. Second, although the search strategy used was thorough, grey literature may not have been captured. Third, the thematic analysis and co-production meetings were systematic and transparent, but the nature of qualitative synthesis is that other teams may have identified other themes or performed categorization in another way. The study team aimed to improve credibility and transferability by performing double coding and within-team discussion, and transparently reporting the methodology. Fourth, the review included studies without surgical patients in the data synthesis. Whilst this may provide opportunities for cross-disciplinary learning, potential differences in the cause of and solutions for sleep disturbance between surgical and other hospitalized patients should be recognized (for example acute postoperative pain).

Improving sleep in hospital for adult surgical patients is likely to require a multimodal strategy, which may include promising components such as environmental modifications, physical sleep adjuvants and aromatherapy. Measures of sleep adopted in published research are heterogenous, and paired objective measurement and patient-reported methods are likely to be important in parallel. There is a lack of high-quality evidence to link sleep improvement with other health-related outcomes and this warrants further exploration in future research.

## Supplementary Material

zrae018_Supplementary_Data

## Data Availability

Search strategy and included papers available upon request to the study management group.

## References

[1] Walker MP . Cognitive consequences of sleep and sleep loss. Sleep Med 2008;9:S29–S34 18929316 10.1016/S1389-9457(08)70014-5

[2] Mackiewicz M, Shockley KR, Romer MA, Galante RJ, Zimmerman JE, Naidoo N et al Macromolecule biosynthesis: a key function of sleep. Physiol Genomics 2007;31:441–457 17698924 10.1152/physiolgenomics.00275.2006

[3] Wang G, Grone B, Colas D, Appelbaum L, Mourrain P. Synaptic plasticity in sleep: learning, homeostasis and disease. Trends Neurosci 2011;34:452–463 21840068 10.1016/j.tins.2011.07.005 PMC3385863

[4] Opp MR, Toth LA. Neural-immune interactions in the regulation of sleep. Front Biosci 2003;8:d768–d779 12700057 10.2741/1061

[5] Irwin M, McClintick J, Costlow C, Fortner M, White J, Gillin JC. Partial night sleep deprivation reduces natural killer and cellular immune responses in humans. FASEB J 1996;10:643–653 8621064 10.1096/fasebj.10.5.8621064

[6] Nagai M, Hoshide S, Kario K. Sleep duration as a risk factor for cardiovascular disease–a review of the recent literature. Curr Cardiol Rev 2010;6:54–61 21286279 10.2174/157340310790231635 PMC2845795

[7] Raymond I, Ancoli-Israel S, Choinière M. Sleep disturbances, pain and analgesia in adults hospitalized for burn injuries. Sleep Med 2004;5:551–559 15511701 10.1016/j.sleep.2004.07.007

[8] Allvin R, Ehnfors M, Rawal N, Idvall E. Experiences of the postoperative recovery process: an interview study. Open Nurs J 2008;2:1–7 19319214 10.2174/1874434600802010001 PMC2582826

[9] Lassen K, Soop M, Nygren J, Cox PB, Hendry PO, Spies C et al Consensus review of optimal perioperative care in colorectal surgery: enhanced recovery after surgery (ERAS) group recommendations. Arch Surg 2009;144:961–969 19841366 10.1001/archsurg.2009.170

[10] DuBose JR, Hadi K. Improving inpatient environments to support patient sleep. Int J Qual Health Care 2016;28:540–553 27512130 10.1093/intqhc/mzw079

[11] Hu RF, Jiang XY, Chen J, Zeng Z, Chen XY, Li Y et al Non-pharmacological interventions for sleep promotion in the intensive care unit. Cochrane Database Syst Rev 2015;10:CD008808 10.1002/14651858.CD008808.pub2 PMC6517220 26439374

[12] Kehlet H, Rung GW, Callesen T. Postoperative opioid analgesia: time for a reconsideration? J Clin Anesth 1996;8:441–445 8872683 10.1016/0952-8180(96)00131-6

[13] Aparício C, Panin F. Interventions to improve inpatients’ sleep quality in intensive care units and acute wards: a literature review. Br J Nurs 2020;29:770–776 32649254 10.12968/bjon.2020.29.13.770

[14] Hutton B, Salanti G, Caldwell DM, Chaimani A, Schmid CH, Cameron C et al The PRISMA extension statement for reporting of systematic reviews incorporating network meta-analyses of health care interventions: checklist and explanations. Ann Intern Med 2015;162:777–784 26030634 10.7326/M14-2385

[15] Staniszewska S, Brett J, Simera I, Seers K, Mockford C, Goodlad S et al GRIPP2 reporting checklists: tools to improve reporting of patient and public involvement in research. BMJ 2017;358:j3453 28768629 10.1136/bmj.j3453 PMC5539518

[16] Altman DG, Simera I, Hoey J, Moher D, Schulz K. EQUATOR: reporting guidelines for health research. Lancet 2008;371:1149–1150 18395566 10.1016/S0140-6736(08)60505-X

[17] Aksu NT, Erdogan A, Ozgur N. Effects of progressive muscle relaxation training on sleep and quality of life in patients with pulmonary resection. Sleep Breath 2018;22:695–702 29290053 10.1007/s11325-017-1614-2

[18] Alparslan GB, Orsal O, Unsal A. Assessment of sleep quality and effects of relaxation exercise on sleep quality in patients hospitalized in internal medicine services in a university hospital: the effect of relaxation exercises in patients hospitalized. Holist Nurs Pract 2016;30:155–165 27078810 10.1097/HNP.0000000000000147

[19] Bani Younis MK, Hayajneh FA, Alduraidi H. Effectiveness of using eye mask and earplugs on sleep length and quality among intensive care patients: a quasi-experimental study. Int J Nurs Pract 2019;25:e12740 31090172 10.1111/ijn.12740

[20] Bartick MC, Thai X, Schmidt T, Altaye A, Solet JM. Decrease in as-needed sedative use by limiting nighttime sleep disruptions from hospital staff. J Hosp Med 2010;5:E20–E24 19768797 10.1002/jhm.549

[21] Canazei M, Bassa D, Jimenez P, Papousek I, Fink A, Weiss E. Effects of an adjunctive, chronotype-based light therapy in hospitalized patients with severe burnout symptoms—a pilot study. Chronobiol Int 2019;36:993–1004 31068015 10.1080/07420528.2019.1604539

[22] Cho MY, Min ES, Hur MH, Lee MS. Effects of aromatherapy on the anxiety, vital signs, and sleep quality of percutaneous coronary intervention patients in intensive care units. Evid Based Complement Alternat Med 2013;2013:381381 23476690 10.1155/2013/381381 PMC3588400

[23] Chong MS, Tan KT, Tay L, Wong YM, Ancoli-Israel S. Bright light therapy as part of a multicomponent management program improves sleep and functional outcomes in delirious older hospitalized adults. Clin Interv Aging 2013;8:565–572 23723696 10.2147/CIA.S44926 PMC3666546

[24] De Rui M, Middleton B, Sticca A, Gatta A, Amodio P, Skene DJ et al Sleep and circadian rhythms in hospitalized patients with decompensated cirrhosis: effect of light therapy. Neurochem Res 2015;40:284–292 25135598 10.1007/s11064-014-1414-z

[25] Dobing S, Dey A, McAlister F, Ringrose J. Non-pharmacologic interventions to improve sleep of medicine inpatients: a controlled study. J Community Hosp Intern Med Perspect 2017;7:287–295 29147469 10.1080/20009666.2017.1379845 PMC5676797

[26] Ducloux D, Guisado H, Pautex S. Promoting sleep for hospitalized patients with advanced cancer with relaxation therapy: experience of a randomized study. Am J Hosp Palliat Care 2013;30:536–540 22964343 10.1177/1049909112459367

[27] Fakhr-Movahedi A, Mirmohammadkhani M, Ramezani H. Effect of milk-honey mixture on the sleep quality of coronary patients: a clinical trial study. Clin Nutr ESPEN 2018;28:132–135 30390870 10.1016/j.clnesp.2018.08.015

[28] Fan-Lun C, Chung C, Lee EHG, Pek E, Ramsden R, Ethier C et al Reducing unnecessary sedative-hypnotic use among hospitalised older adults. BMJ Qual Saf 2019;28:1039–1045 10.1136/bmjqs-2018-009241 31270252

[29] Faraklas I, Holt B, Tran S, Lin H, Saffle J, Cochran A. Impact of a nursing-driven sleep hygiene protocol on sleep quality. J Burn Care Res 2013;34:249–254 23412331 10.1097/BCR.0b013e318283d175

[30] Farrehi PM, Clore KR, Scott JR, Vanini G, Clauw DJ. Efficacy of sleep tool education during hospitalization: a randomized controlled trial. Am J Med 2016;129:1329.e9–1329.e17 10.1016/j.amjmed.2016.08.001 27566502

[31] Garcia MK, Cohen L, Spano M, Spelman A, Hashmi Y, Chaoul A et al Inpatient acupuncture at a major cancer center. Integr Cancer Ther 2018;17:148–152 28050924 10.1177/1534735416685403 PMC5950949

[32] Gardner G, Collins C, Osborne S, Henderson A, Eastwood M. Creating a therapeutic environment: a non-randomised controlled trial of a quiet time intervention for patients in acute care. Int J Nurs Stud 2009;46:778–786 19167711 10.1016/j.ijnurstu.2008.12.009

[33] Gathecha E, Rios R, Buenaver LF, Landis R, Howell E, Wright S. Pilot study aiming to support sleep quality and duration during hospitalizations. J Hosp Med 2016;11:467–472 26970217 10.1002/jhm.2578

[34] Giménez MC, Geerdinck LM, Versteylen M, Leffers P, Meekes GJ, Herremans H et al Patient room lighting influences on sleep, appraisal and mood in hospitalized people. J Sleep Res 2017;26:236–246 27862514 10.1111/jsr.12470

[35] Hajibagheri A, Babaii A, Adib-Hajbaghery M. Effect of *Rosa damascene* aromatherapy on sleep quality in cardiac patients: a randomized controlled trial. Complement Ther Clin Pract 2014;20:159–163 25129884 10.1016/j.ctcp.2014.05.001

[36] Henriksen TEG, Grønli J, Assmus J, Fasmer OB, Schoeyen H, Leskauskaite I et al Blue-blocking glasses as additive treatment for mania: effects on actigraphy-derived sleep parameters. J Sleep Res 2020;29:e12984 31967375 10.1111/jsr.12984

[37] Jones C, Dawson D. Eye masks and earplugs improve patient's perception of sleep. Nurs Crit Care 2012;17:247–254 22897811 10.1111/j.1478-5153.2012.00501.x

[38] Kuon C, Wannier R, Harrison J, Tague C. Massage for symptom management in adult inpatients with hematologic malignancies. Glob Adv Health Med 2019;8:2164956119849390 10.1177/2164956119849390 PMC6509973 31106038

[39] Lareau R, Benson L, Watcharotone K, Manguba G. Examining the feasibility of implementing specific nursing interventions to promote sleep in hospitalized elderly patients. Geriatr Nurs 2008;29:197–206 18555161 10.1016/j.gerinurse.2007.10.020

[40] Le Guen M, Nicolas-Robin A, Lebard C, Arnulf I, Langeron O. Earplugs and eye masks vs routine care prevent sleep impairment in post-anaesthesia care unit: a randomized study. Br J Anaesth 2014;112:89–95 24172057 10.1093/bja/aet304

[41] Lee KA, Gay CL. Improving sleep for hospitalized antepartum patients: a non-randomized controlled pilot study. J Clin Sleep Med 2017;13:1445–1453 29117884 10.5664/jcsm.6846 PMC5695992

[42] Li SY, Wang TJ, Vivienne Wu SF, Liang SY, Tung HH. Efficacy of controlling night-time noise and activities to improve patients’ sleep quality in a surgical intensive care unit. J Clin Nurs 2011;20:396–407 21219521 10.1111/j.1365-2702.2010.03507.x

[43] Locke CL, Pope DS. Assessment of medical-surgical patients’ perception of hospital noises and reported ability to rest. Clin Nurse Spec 2017;31:261–267 28806232 10.1097/NUR.0000000000000321

[44] Lytle J, Mwatha C, Davis KK. Effect of lavender aromatherapy on vital signs and perceived quality of sleep in the intermediate care unit: a pilot study. Am J Crit Care 2014;23:24–29 24382614 10.4037/ajcc2014958

[45] Maidl CA, Leske JS, Garcia AE. The influence of “quiet time” for patients in critical care. Clin Nurs Res 2014;23:544–559 23847172 10.1177/1054773813493000

[46] McDowell JA, Mion LC, Lydon TJ, Inouye SK. A nonpharmacologic sleep protocol for hospitalized older patients. J Am Geriatr Soc 1998;46:700–705 9625184 10.1111/j.1532-5415.1998.tb03803.x

[47] Menger J, Urbanek B, Skhirtladze-Dworschak K, Wolf V, Fischer A, Rinösl H et al Earplugs during the first night after cardiothoracic surgery may improve a fast-track protocol. Minerva Anestesiol 2018;84:49–57 28726359 10.23736/S0375-9393.17.11758-X

[48] Nooner AK, Dwyer K, DeShea L, Yeo TP. Using relaxation and guided imagery to address pain, fatigue, and sleep disturbances: a pilot study. Clin J Oncol Nurs 2016;20:547–552 27668375 10.1188/16.CJON.547-552

[49] Norton C, Flood D, Brittin A, Miles J. Improving sleep for patients in acute hospitals. Nurs Stand 2015;29:35–42 10.7748/ns.29.28.35.e8947 25758517

[50] Okkels N, Jensen LG, Skovshoved LC, Arendt R, Blicher AB, Vieta E et al Lighting as an aid for recovery in hospitalized psychiatric patients: a randomized controlled effectiveness trial. Nord J Psychiatry 2020;74:105–114 31603013 10.1080/08039488.2019.1676465

[51] Olson DM, Borel CO, Laskowitz DT, Moore DT, McConnell ES. Quiet time: a nursing intervention to promote sleep in neurocritical care units. Am J Crit Care 2001;10:74–78 11244674

[52] Ong TL, Ruppert MM, Akbar M, Rashidi P, Ozrazgat-Baslanti T, Bihorac A et al Improving the intensive care patient experience with virtual reality–a feasibility study. Crit Care Explor 2020;2:e0122 32695991 10.1097/CCE.0000000000000122 PMC7314318

[53] Ozlu ZK, Bilican P. Effects of aromatherapy massage on the sleep quality and physiological parameters of patients in a surgical intensive care unit. Afr J Tradit Complement Altern Med 2017;14:83–88 10.21010/ajtcam.v14i3.9 PMC5412241 28480419

[54] Patel J, Baldwin J, Bunting P, Laha S. The effect of a multicomponent multidisciplinary bundle of interventions on sleep and delirium in medical and surgical intensive care patients. Anaesthesia 2014;69:540–549 24813132 10.1111/anae.12638

[55] Pati D, Freier P, O'Boyle M, Amor C, Valipoor S. The impact of simulated nature on patient outcomes: a study of photographic sky compositions. HERD 2016;9:36–51 26199272 10.1177/1937586715595505

[56] Pattison HM, Robertson CE. The effect of ward design on the well-being of post-operative patients. J Adv Nurs 1996;23:820–826 8675902 10.1111/j.1365-2648.1996.tb00056.x

[57] Richards KC . Effect of a back massage and relaxation intervention on sleep in critically ill patients. Am J Crit Care 1998;7:288–299 9656043

[58] Richardson S . Effects of relaxation and imagery on the sleep of critically ill adults. Dimens Crit Care Nurs 2003;22:182–190 12893996 10.1097/00003465-200307000-00009

[59] Ryu MJ, Park JS, Park H. Effect of sleep-inducing music on sleep in persons with percutaneous transluminal coronary angiography in the cardiac care unit. J Clin Nurs 2012;21:728–735 22082250 10.1111/j.1365-2702.2011.03876.x

[60] Scarpa M, Pinto E, Saraceni E, Cavallin F, Parotto M, Alfieri R et al Randomized clinical trial of psychological support and sleep adjuvant measures for postoperative sleep disturbance in patients undergoing oesophagectomy. Br J Surg 2017;104:1307–1314 28707741 10.1002/bjs.10609

[61] Scotto CJ, McClusky C, Spillan S, Kimmel J. Earplugs improve patients’ subjective experience of sleep in critical care. Nurs Crit Care 2009;14:180–184 19531035 10.1111/j.1478-5153.2009.00344.x

[62] Silvius-Byron SA, Florimonte C, Panganiban EG, Ulmer JF. What is the best?: simple versus visitor restricted rest period. J Nurs Adm 2014;44:291–297 24759202 10.1097/NNA.0000000000000069

[63] Smith MC, Kemp J, Hemphill L, Vojir CP. Outcomes of therapeutic massage for hospitalized cancer patients. J Nurs Scholarsh 2002;34:257–262 12237988 10.1111/j.1547-5069.2002.00257.x

[64] Spence J, Murray T, Tang AS, Butler RS, Albert NM. Nighttime noise issues that interrupt sleep after cardiac surgery. J Nurs Care Qual 2011;26:88–95 20683198 10.1097/NCQ.0b013e3181ed939a

[65] Su CP, Lai HL, Chang ET, Yiin LM, Perng SJ, Chen PW. A randomized controlled trial of the effects of listening to non-commercial music on quality of nocturnal sleep and relaxation indices in patients in medical intensive care unit. J Adv Nurs 2013;69:1377–1389 22931483 10.1111/j.1365-2648.2012.06130.x

[66] Sweity S, Finlay A, Lees C, Monk A, Sherpa T, Wade D. SleepSure: a pilot randomized-controlled trial to assess the effects of eye masks and earplugs on the quality of sleep for patients in hospital. Clin Rehabil 2019;33:253–261 30322272 10.1177/0269215518806041

[67] Tas D, Uncu D, Sendur MA, Koca N, Zengin N. Acupuncture as a complementary treatment for cancer patients receiving chemotherapy. Asian Pac J Cancer Prev 2014;15:3139–3144 24815460 10.7314/apjcp.2014.15.7.3139

[68] Thomas KP, Salas RE, Gamaldo C, Chik Y, Huffman L, Rasquinha R et al Sleep rounds: a multidisciplinary approach to optimize sleep quality and satisfaction in hospitalized patients. J Hosp Med 2012;7:508–512 22407674 10.1002/jhm.1934

[69] Van Rompaey B, Elseviers MM, Van Drom W, Fromont V, Jorens PG. The effect of earplugs during the night on the onset of delirium and sleep perception: a randomized controlled trial in intensive care patients. Crit Care 2012;16:R73 22559080 10.1186/cc11330 PMC3580615

[70] Vitinius F, Hellmich M, Matthies A, Bornkessel F, Burghart H, Albus C et al Feasibility of an interval, inspiration-triggered nocturnal odorant application by a novel device: a patient-blinded, randomised crossover, pilot trial on mood and sleep quality of depressed female inpatients. Eur Arch Otorhinolaryngol 2014;271:2443–2454 24390040 10.1007/s00405-013-2873-6

[71] Wakamura T, Tokura H. Influence of bright light during daytime on sleep parameters in hospitalized elderly patients. J Physiol Anthropol Appl Human Sci 2001;20:345–351 10.2114/jpa.20.345 11840687

[72] Yazdannik AR, Zareie A, Hasanpour M, Kashefi P. The effect of earplugs and eye mask on patients’ perceived sleep quality in intensive care unit. Iran J Nurs Midwifery Res 2014;19:673–678 25558268 PMC4280735

[73] van den Ende ES, Merten H, Van der Roest L, Toussaint B, van Rijn Q, Keesenberg M et al Evaluation of nonpharmacologic interventions and sleep outcomes in hospitalized medical and surgical patients: a nonrandomized controlled trial. JAMA Netw Open 2022;5:e2232623 36129708 10.1001/jamanetworkopen.2022.32623 PMC9494194

[74] Obanor OO, McBroom MM, Elia JM, Ahmed F, Sasaki JD, Murphy KM et al The impact of earplugs and eye masks on sleep quality in surgical ICU patients at risk for frequent awakenings. Crit Care Med 2021;49:e822–e832 33870919 10.1097/CCM.0000000000005031

[75] Leong RW, Davies LJ, Fook-Chong S, Ng SY, Lee YL. Effect of the use of earplugs and eye masks on the quality of sleep after major abdominal surgery: a randomised controlled trial. Anaesthesia 2021;76:1482–1491 33881774 10.1111/anae.15468

[76] Tranmer JE, Minard J, Fox LA, Rebelo L. The sleep experience of medical and surgical patients. Clin Nurs Res 2003;12:159–173 12741668 10.1177/1054773803012002004

[77] Freedman NS, Kotzer N, Schwab RJ. Patient perception of sleep quality and etiology of sleep disruption in the intensive care unit. Am J Respir Crit Care Med 1999;159:1155–1162 10194160 10.1164/ajrccm.159.4.9806141

[78] Casarett D, Karlawish JH, Sugarman J. Determining when quality improvement initiatives should be considered research: proposed criteria and potential implications. JAMA 2000;283:2275–2280 10807388 10.1001/jama.283.17.2275

[79] O'Cathain A, Croot L, Duncan E, Rousseau N, Sworn K, Turner KM et al Guidance on how to develop complex interventions to improve health and healthcare. BMJ Open 2019;9:e029954–e029954 10.1136/bmjopen-2019-029954 PMC6701588 31420394

[80] van Maanen A, Meijer AM, van der Heijden KB, Oort FJ. The effects of light therapy on sleep problems: a systematic review and meta-analysis. Sleep Med Rev 2016;29:52–62 26606319 10.1016/j.smrv.2015.08.009

[81] Kleif J, Waage J, Christensen KB, Gögenur I. Systematic review of the QoR-15 score, a patient-reported outcome measure measuring quality of recovery after surgery and anaesthesia. Br J Anaesth 2018;120:28–36 29397134 10.1016/j.bja.2017.11.013

[82] Beecroft JM, Ward M, Younes M, Crombach S, Smith O, Hanly PJ. Sleep monitoring in the intensive care unit: comparison of nurse assessment, actigraphy and polysomnography. Intensive Care Med 2008;34:2076–2083 18521566 10.1007/s00134-008-1180-y

[83] De Zambotti M . Wearable sleep technology in clinical and research settings. Med Sci Sports Exercise 2019;51:1538–1558 10.1249/MSS.0000000000001947 PMC6579636 30789439

[84] Stea S, Beraudi A, De Pasquale D. Essential oils for complementary treatment of surgical patients: state of the art. Evid Based Complement Alternat Med 2014;2014:726341–6 24707312 10.1155/2014/726341 PMC3953654

