# Supplementary material for: Non-pharmacological interventions to improve sleep quality and quantity for hospitalized adult patients—co-produced study with surgical patient partners: systematic review
Source: BJS Open. 2024 Apr 10;8(2):zrae018. doi: 10.1093/bjsopen/zrae018 (PMC11004792; doi:10.1093/bjsopen/zrae018)
Supplement: zrae018_Supplementary_Data [file zrae018_supplementary_data.docx]

**Non-pharmacological interventions to improve sleep quality and quantity for hospitalised adult patients – systematic review: a co-produced study with surgical patient partners**

Radhika Acharya^1^, Sue Blackwell^2^, Joana Simoes^1^, Benjamin Harris^1^, Lesley Booth^3^, Aneel Bhangu^1^, James Glasbey^1^

*^1^NIHR Global Health Research Unit on Global Surgery,* *University of Birmingham, Institute of Translation Medicine, Birmingham, United Kingdom.*

*^2^Patient Liaison Group (PLG), Association of Coloproctology of Great Britain and Ireland, London, United Kingdom.*

*^3^Patients and Researchers Together (PART), Bowel Research United Kingdom, London, United Kingdom.*

**Corresponding author:** Mr James Glasbey MBBCh MRCS, NIHR Doctoral Research Fellow in Global Surgery, NIHR Global Health Research Unit on Global Surgery, University of Birmingham, Institute of Translational Medicine, Mindelsohn Way, Birmingham, B15 2TH, Email: [j.glasbey@bham.ac.uk](mailto:j.glasbey@bham.ac.uk)

**Competing interests:** No conflicts of interest are declared.

**Data Availability Statement:** Search strategy and included papers available upon request to study management group.

**Supplementary Method and Tables- Index**

| **Supplementary Method** | |  |  |
| --- | --- | --- | --- |
| Detail | | *pag. 2* |  |
| **Supplementary Table S1** | |  |  |
| Detail | | *pag. 5* |  |
| **Supplementary Table S2** | |  |  |
| Detail | | *pag. 6* |  |
|  | |  |  |
|  | |  |  |
| **Supplementary Method – Search Strategy**   \| **Adult** \| **Sleep** \| **Hospital** \| \| --- \| --- \| --- \| \| ((("Adult"[Mesh] OR "adult"[All Fields] OR "adults"[All Fields] OR "aged"[All Fields[ OR "elderly"[All Fields] OR "aged, 80 and over"[All Fields] OR "oldest old"[All Fields] OR "nonagenarian"[All Fields] OR "nonageniarnans"[All Fields] OR "octogenarian"[All Fields] OR "octogenarians"[All Fields] OR "centenarian"[All Fields] OR "centernarians"[All Fields] OR "frail elderly"[All Fields] OR "frail elder"[All Fields] OR "frail elders"[All Fields] OR "functionally-impaired elderly"[All Fields] OR "frail older adult"[All Fields] OR "frail older adults"[All Fields] OR "middle aged"[All Fields] OR "middle age"[All Fields] OR "young adult"[All Fields] OR "young adults"[All Fields] OR "aged hospital patient"[All Fields] OR "aged hospital patients"[All Fields] OR "aged hospitalized"[All Fields] OR "very elderly"[All Fields] OR "very old"[All Fields] OR "aged patient"[All Fields] OR "aged patients"[All Fields] OR "aged people"[All Fields] OR "elderly patient"[All Fields] OR "elderly patients"[All Fields] OR "elderly people"[All Fields] OR "senior citizen"[All Fields] OR "senior citizens"[All Fields] OR "senium"[All Fields])) \| ((("Sleep"[Mesh:noexp] OR "Sleep"[All Fields] OR "Sleep Deprivation"[Mesh] OR "Sleep Deprivation"[All Fields] OR "Sleep Deprivations"[All Fields] OR "REM Sleep Deprivation"[All Fields] OR "REM Sleep Deprivations"[All Fields] OR "Sleep Fragmentation"[All Fields] OR "Sleep Fragmentations"[All Fields] OR "Insufficient Sleep Syndrome"[All Fields] OR "Sleep Stages"[Mesh] OR "Sleep Stages"[All Fields] OR "Sleep Stage"[All Fields] OR "Drowsiness"[All Fields] OR "REM Sleep"[All Fields] OR "Sleep, REM"[All Fields] OR "Rhombencephalic Sleep"[All Fields] OR "Fast Wave Sleep"[All Fields] OR "Fast-wave Sleep"[All Fields] OR "Paradoxical Sleep"[All Fields] OR "Rapid Eye Movements"[All Fields] OR "Rapid Eye Movement"[All Fields] OR "Slow Wave Sleep"[All Fields] OR "Slow-wave Sleep"[All Fields] OR "sleep induction"[All Fields] OR "sleep time"[All Fields] OR "sleeping time"[All Fields] OR "Disorders of Excessive Somnolence"[Mesh] OR "disorders of excessive somnolence"[All Fields] OR "somnolence"[All Fields] OR "hypersomnolence"[All Fields] OR "hypersomnia"[All Fields] OR "hypersomnias"[All Fields] OR "diurnal sleepiness"[All Fields] OR "sleepiness"[All Fields] OR "Sleep Stages"[Mesh] OR "nonREM sleep"[All Fields] OR "non REM sleep"[All Fields] OR "daytime somnolence"[All Fields] OR "daytime sleepiness"[All Fields] OR "sleep latency"[All Fields] OR "sleeping"[All Fields] OR "night sleep"[All Fields] OR "nocturnal sleep"[All Fields] OR "rapid eye movement deprivation"[All Fields] OR "daytime sleepiness"[All Fields] OR "delta sleep"[All Fields] OR "stage 3 sleep"[All Fields] OR "slow sleep"[All Fields] OR "activated sleep"[All Fields] OR "deep sleep"[All Fields] OR "desynchronized sleep"[All Fields])) OR (("fast sleep"[All Fields] OR "low voltage sleep"[All Fields] OR "low voltage fast sleep"[All Fields] OR "low voltage fast sleep"[All Fields] OR "paradoxal sleep"[All Fields] OR "paradoxic sleep"[All Fields] OR "parasleep"[All Fields] OR "rapid eye movement sleep"[All Fields] OR "REM phase"[All Fields] OR "stage 5 sleep"[All Fields] OR "Sleep Initiation and Maintenance Disorders"[Mesh:NoExp] OR "Sleep Initiation and Maintenance Disorders"[All Fields] OR "sleep initiation and maintenance disorder"[All Fields] OR "Disorders of Initiating and Maintaining Sleep"[All Fields] OR "DIMS"[All Fields] OR "Early Awakening"[All Fields] OR "Nonorganic Insomnia"[All Fields] OR "Nonorganic insomnias"[All Fields] OR "Primary Insomnia"[All Fields] OR "primary insomnias"[All Fields] OR "Transient Insomnia"[All Fields] OR "transient insomnias"[All Fields] OR "Rebound Insomnia"[All Fields] OR "rebound insomnias"[All Fields] OR "Secondary Insomnia"[All Fields] OR "secondary insomnias"[All Fields] OR "sleep initiation dysfunctions"[All Fields] OR "Sleeplessness"[All Fields] OR "Insomnia Disorder"[All Fields] OR "Insomnia Disorders"[All Fields] OR "Insomnia"[All Fields] OR "Insomnias"[All Fields] OR "Chronic Insomnia"[All Fields] OR "chronic insomnias"[All Fields] OR "psychophysiological insomnia"[All Fields] OR "psychophysiological insomnias"[All Fields] OR "agrypnia"[All Fields] OR "agrypnias"[All Fields] OR "hyposomnia"[All Fields] OR "hyposomnias"[All Fields]) \| ((("Hospitalization"[Mesh] OR "hospitalization"[All Fields] OR "hospitalizations"[All Fields] OR "hospitalized"[All Fields] OR "length of stay"[All Fields] OR "stay length"[All Fields] OR "stay lengths"[All Fields] OR "patient admission"[All Fields] OR "patient admissions"[All Fields] OR "voluntary admission"[All Fields] OR "voluntary admissions"[All Fields] OR "patient discharge"[All Fields] OR "patient discharges"[All Fields] OR "discharge planning"[All Fields] OR "discharge plannings"[All Fields] OR "patient readmission"[All Fields] OR "patient readmissions"[All Fields] OR "hospital readmissions"[All Fields] OR "hospital readmission"[All Fields] OR "readmission"[All Fields] OR "readmissions"[All Fields] OR "patient transfer"[All Fields] OR "patient transfers"[All Fields] OR "patient dumping"[All Fields] OR "hospital patient"[All Fields] OR "hospital patients"[All Fields] OR "Inpatients"[Mesh] OR "inpatient"[All Fields] OR "inpatients"[All Fields] OR "hospitalised patient"[All Fields] OR "hospitalised patients"[All Fields])) OR (("hospitalized patient"[All Fields] OR "hospitalized patients"[All Fields] OR "in-hospital patient"[All Fields] OR "in-hospital patients"[All Fields] OR "in-patient"[All Fields] OR "aged hospital patient"[All Fields] OR "aged hospital patients"[All Fields] OR "aged hospitalized"[All Fields] OR "hospital admission"[All Fields] OR "hospital admissions"[All Fields] OR "hospital admittance"[All Fields] OR "hospital admittances"[All Fields] OR "hospital discharge"[All Fields] OR "hospital discharges"[All Fields] OR "patient transport"[All Fields] OR "intrahospital transfer"[All Fields] OR "hospital stay"[All Fields] OR "short stay hospitalization"[All Fields] OR "short stay hospitalizations"[All Fields] OR "night care"[Mesh] OR "night care"[All Fields]))) \|   **Table S1.** Guidance for Reporting Involvement of Patients and the Public (GRIPP2) short-form reporting checklist used for patient and public involvement (PPI) | |  |  |
| **Selection and topic** | **Item** | | |
| 1: Aim | The aim of PPI in the current scoping review is to develop consensus and refine themes identified for non-pharmacological interventions to improve sleep quality and quantity. | | |
| 2: Methods | Once full text screening had been completed, a patient representative was recruited to assist at all stages of thematic analysis. The patient representative helped refine the themes gained from data collection. | | |
| 3: Study results | PPI contributed to the scoping review in several ways, including:   - The patient partner helped narrow down the initial 17 themes into 2 themes, which were also understandable and appropriate for the lay person. - The patient representative had experienced sleep problems during hospitalisation and shed light on their experience on the major contributors to sleep disruption, such as care activities. - Following a thorough discussion of the existing interventions, the patient representative offered alternate solutions for improving sleep during hospitalisation, which weren’t evident from the existing studies of the scoping review. | | |
| 4: Discussion and conclusions | Patient and public involvement in this scoping review played a vital role during theming of interventions as well as providing direct insight into the extent of sleep disruption that occurs in hospital. This may have been due to several factors. Firstly, the patient partner had previous experience of hospitalisation and the sleep disruptions that occurred during their stay. Secondly, the patient partner was involved at the correct stage of the scoping review and were already actively involved in patient and public involvement projects elsewhere. | | |
| 5: Reflections/critical perspective | The PPI was embedded during the methods part of the review and the active collaboration between the research time and patient partner worked extremely well. As gaps in literature regarding alternative interventions were brough forward during the PPI meeting, it would be useful for primary studies to include patient collaboration earlier on and as part of the research team, | | |

**Table S2.** Summary of included studies of non-pharmacological interventions in adult inpatients

| **Article (First author and year)** | **Country** | **Sample size** | **Setting** | **Participant description** | **Mean patient age** | **Study design** |
| --- | --- | --- | --- | --- | --- | --- |
| Aksu. 2018 [1] | Turkey | 26 | Thoracic surgery clinic | Surgical | >50 | RCT |
| Alparslan. 2016 [2] | USA | 282 | General medicine ward | Medical | >50 | Non-randomised interventional trial |
| Bani Younis. 2019 [3] | Jordan | 103 | Medical ICU | Medical | >50 | Prospective cohort |
| Bartick. 2010 [4] | USA | 106 | Medical-surgical unit | Medical and surgical | >60 | Prospective cohort |
| Canazei. 2019 [5] | Austria | 56 | Psychosomatic clinic | Psychiatric | <50 | RCT |
| Cho. 2013 [6] | South Korea | 56 | ICU | Medical | >50 | Non-randomised interventional trial |
| Chong. 2013 [7] | Singapore | 228 | Geriatric unit | Medical | >70 | Prospective cohort |
| De Rui. 2015 [8] | Italy | 12 | Liver unit | Medical | >50 | Prospective cohort |
| Dobing. 2017 [9] | Canada | 81 | General Internal Medicine Ward | Medical | Not reported | Non-randomised interventional trial |
| Ducloux. 2013 [10] | Switzerland | 18 | Oncology patients | Medical | >60 | RCT |
| Fakhr-Movahedi. 2018 [11] | Iran | 68 | Coronary care unit | Medical | >60 | RCT |
| Fan-Lun, 2019 [12] | Canada | 7692 | General Internal Medicine, Cardiology and Surgical Ward | Medical and surgical | Not reported | Prospective cohort |
| Faraklas. 2013 [13] | USA | 130 | Burn-trauma ICU | Surgical | Not reported | Prospective cohort |
| Farrehi, 2016 [14] | USA | 120 | Cardiac unit | Medical | >50 | RCT |
| Garcia. 2018 [15] | USA | 172 | Oncology unit | Medical | Not reported | Prospective cohort |
| Gardner. 2009 [16] | Australia | 299 | Orthopaedic Ward | Surgical | >50 | Non-randomised interventional trial |
| Gathecha. 2016 [17] | USA | 112 | General medicine ward | Medical | >50 | Prospective cohort |
| Gimenez. 2017 [18] | Netherlands | 196 | Cardiology ward | Medical | >60 | Non-randomised interventional trial |
| Hajibagheri. 2014 [19] | Iran | 60 | Cardiac unit | Medical and surgical | >60 | RCT |
| Henriksen. 2020 [20] | Norway | 32 | Psychiatric clinic | Psychiatric | <50 | RCT |
| Jones. 2012 [21] | UK | 100 | Medical and surgical ICU | Medical and surgical | >50 | Prospective cohort |
| Kuon. 2019 [22] | USA | 109 | Bone Marrow Transplant Unit | Medical | >50 | Prospective cohort |
| Lareau. 2008 [23] | USA | 59 | General medical and cardiology unit | Medical | >70 | RCT |
| Le Guen. 2014 [24] | France | 46 | Post-anaesthesia ward | Surgical | Not reported | RCT |
| Lee. 2017 [25] | USA | 25 | Antepartum Unit | Medical | <50 | Non-randomised interventional trial |
| Leong. 2021[26] | Singapore | 93 | Colorectal surgery ward | Surgical | >50 | RCT |
| Li. 2011 [27] | China | 69 | Surgical ICU | Surgical | >50 | RCT |
| Locke. 2017 [28] | USA | 28 | Medical-surgical unit | Medical and surgical | >60 | Non-randomised interventional trial |
| Lytle. 2014 [29] | USA | 50 | Intermediate care unit | Medical | >50 | RCT |
| Maidl. 2014 [30] | USA | 129 | Neurosciences ICU and cardiovascular ICU | Medical | >50 | Prospective cohort |
| McDowell. 1998 [31] | USA | 111 | General Medical unit | Medical | >70 | Prospective cohort |
| Menger. 2018 [32] | Austria | 63 | Cardiothoracic Unit | Surgical | >70 | RCT |
| Nooner. 2016 [33] | USA | 12 | Oncology unit | Medical | Not reported | RCT |
| Norton. 2015 [34] | UK | 783 | Medical and surgical wards | Medical and surgical | Not reported | Prospective cohort |
| Obanor. 2021[35] | USA | 87 | Surgical ICU | Surgical | >50 | RCT |
| Okkels. 2020 [36] | Denmark | 54 | Psychiatric ward | Psychiatric | <50 | RCT |
| Olson. 2001 [37] | USA | 239 | Neurocritical care unit | Medical and surgical | Not reported | Prospective cohort |
| Ong. 2020 [38] | USA | 46 | Trauma and surgical ICU | Medical and surgical | >50 | Prospective cohort |
| Ozlu. 2017 [39] | Turkey | 60 | Surgical ICU | Surgical | Not reported | Non-randomised interventional trial |
| Patel. 2014 [40] | UK | 338 | Medical and surgical ICU | Medical and surgical | Not reported | Prospective cohort |
| Pati. 2016 [41] | USA | 181 | Medical-surgical unit | Medical and surgical | >50 | RCT |
| Pattison. 1996 [42] | UK | 68 | Medical-surgical unit | Surgical | Not reported | Prospective cohort |
| Richards. 1998 [43] | USA | 69 | ICU | Medical | >50 | RCT |
| Richardson. 2003 [44] | USA | 36 | ICU | Medical and surgical | >50 | RCT |
| Ryu. 2012 [45] | South Korea | 58 | Cardiac care unit | Medical | >60 | RCT |
| Scarpa. 2017 [46] | Italy | 87 | Digestive Tract Surgical Unit | Medical | >60 | RCT |
| Scotto. 2009 [47] | USA | 88 | Medical and surgical ICU | Medical and surgical | >60 | RCT |
| Silvius-Byron. 2014 [48] | USA | 52 | Intermediate care unit | Medical and surgical | >70 | RCT |
| Smith. 2002 [49] | USA | 41 | Oncology unit | Medical | >60 | Non-randomised interventional trial |
| Spence. 2011 [50] | USA | 37 | Cardiac surgery ward | Surgical | >60 | Prospective cohort |
| Su. 2013 [51] | Taiwan | 28 | Medical ICU | Medical | >60 | RCT |
| Sweity. 2019 [52] | UK | 206 | Medical and surgical ward | Medical and surgical | >50 | RCT |
| Tas. 2014 [53] | Turkey | 45 | Oncology Unit | Medical | >50 | Prospective cohort |
| Thomas. 2012 [54] | USA | 95 | Neurological ward | Medical | <50 | Prospective cohort |
| Van den Ende. 2022[55] | Netherlands | 374 | Medical and surgical wards | Medical and surgical | >50 | Non-randomised interventional trial |
| Van Rompaey. 2012 [56] | Belgium | 136 | Medical ICU | Medical | >50 | RCT |
| Vitinius. 2014 [57] | Germany | 27 | Psychiatric unit | Psychiatric | <50 | RCT |
| Wakamura. 2001 [58] | Japan | 7 | Chest disease ward | Medical | >50 | Prospective cohort |
| Yazdannik. 2014 [59] | Iran | 50 | Medical ICU | Medical | Not reported | RCT |

**References**

1. Aksu, N.T., A. Erdogan, and N. Ozgur, *Effects of progressive muscle relaxation training on sleep and quality of life in patients with pulmonary resection.* Sleep Breath, 2018. **22**(3): p. 695-702.

2. Alparslan, G.B., O. Orsal, and A. Unsal, *Assessment of Sleep Quality and Effects of Relaxation Exercise on Sleep Quality in Patients Hospitalized in Internal Medicine Services in a University Hospital: The Effect of Relaxation Exercises in Patients Hospitalized.* Holist Nurs Pract, 2016. **30**(3): p. 155-65.

3. Bani Younis, M.K., F.A. Hayajneh, and H. Alduraidi, *Effectiveness of using eye mask and earplugs on sleep length and quality among intensive care patients: A quasi-experimental study.* Int J Nurs Pract, 2019. **25**(3): p. e12740.

4. Bartick, M.C., et al., *Decrease in as-needed sedative use by limiting nighttime sleep disruptions from hospital staff.* J Hosp Med, 2010. **5**(3): p. E20-4.

5. Canazei, M., et al., *Effects of an adjunctive, chronotype-based light therapy in hospitalized patients with severe burnout symptoms - a pilot study.* Chronobiol Int, 2019. **36**(7): p. 993-1004.

6. Cho, M.Y., et al., *Effects of aromatherapy on the anxiety, vital signs, and sleep quality of percutaneous coronary intervention patients in intensive care units.* Evid Based Complement Alternat Med, 2013. **2013**: p. 381381.

7. Chong, M.S., et al., *Bright light therapy as part of a multicomponent management program improves sleep and functional outcomes in delirious older hospitalized adults.* Clin Interv Aging, 2013. **8**: p. 565-72.

8. De Rui, M., et al., *Sleep and circadian rhythms in hospitalized patients with decompensated cirrhosis: effect of light therapy.* Neurochem Res, 2015. **40**(2): p. 284-92.

9. Dobing, S., et al., *Non-pharmacologic interventions to improve sleep of medicine inpatients: a controlled study.* J Community Hosp Intern Med Perspect, 2017. **7**(5): p. 287-295.

10. Ducloux, D., H. Guisado, and S. Pautex, *Promoting sleep for hospitalized patients with advanced cancer with relaxation therapy: experience of a randomized study.* Am J Hosp Palliat Care, 2013. **30**(6): p. 536-40.

11. Fakhr-Movahedi, A., M. Mirmohammadkhani, and H. Ramezani, *Effect of milk-honey mixture on the sleep quality of coronary patients: A clinical trial study.* Clin Nutr ESPEN, 2018. **28**: p. 132-135.

12. Fan-Lun, C., et al., *Reducing unnecessary sedative-hypnotic use among hospitalised older adults.* BMJ Quality & Safety. **28**(12): p. 1039-1045.

13. Faraklas, I., et al., *Impact of a nursing-driven sleep hygiene protocol on sleep quality.* J Burn Care Res, 2013. **34**(2): p. 249-54.

14. Farrehi, P.M., et al., *Efficacy of Sleep Tool Education During Hospitalization: A Randomized Controlled Trial.* American Journal of Medicine. **129**(12): p. 1329.e9-1329.e17.

15. Garcia, M.K., et al., *Inpatient Acupuncture at a Major Cancer Center.* Integrative Cancer Therapies. **17**(1): p. 148-152.

16. Gardner, G., et al., *Creating a therapeutic environment: a non-randomised controlled trial of a quiet time intervention for patients in acute care.* Int J Nurs Stud, 2009. **46**(6): p. 778-86.

17. Gathecha, E., et al., *Pilot study aiming to support sleep quality and duration during hospitalizations.* J Hosp Med, 2016. **11**(7): p. 467-72.

18. Gimenez, M.C., et al., *Patient room lighting influences on sleep, appraisal and mood in hospitalized people.* J Sleep Res, 2017. **26**(2): p. 236-246.

19. Hajibagheri, A., A. Babaii, and M. Adib-Hajbaghery, *Effect of Rosa damascene aromatherapy on sleep quality in cardiac patients: a randomized controlled trial.* Complement Ther Clin Pract, 2014. **20**(3): p. 159-63.

20. Henriksen, T.E.G., et al., *Blue-blocking glasses as additive treatment for mania: Effects on actigraphy-derived sleep parameters.* J Sleep Res, 2020. **29**(5): p. e12984.

21. Jones, C. and D. Dawson, *Eye masks and earplugs improve patient's perception of sleep.* Nurs Crit Care, 2012. **17**(5): p. 247-54.

22. Kuon, C., et al., *Massage for Symptom Management in Adult Inpatients With Hematologic Malignancies.* Glob Adv Health Med, 2019. **8**: p. 2164956119849390.

23. Lareau, R., et al., *Examining the feasibility of implementing specific nursing interventions to promote sleep in hospitalized elderly patients.* Geriatr Nurs, 2008. **29**(3): p. 197-206.

24. Le Guen, M., et al., *Earplugs and eye masks vs routine care prevent sleep impairment in post-anaesthesia care unit: a randomized study.* Br J Anaesth, 2014. **112**(1): p. 89-95.

25. Lee, K.A. and C.L. Gay, *Improving Sleep for Hospitalized Antepartum Patients: A Non-Randomized Controlled Pilot Study.* J Clin Sleep Med, 2017. **13**(12): p. 1445-1453.

26. Leong, R.W., et al., *Effect of the use of earplugs and eye masks on the quality of sleep after major abdominal surgery: a randomised controlled trial.* Anaesthesia, 2021. **76**(11): p. 1482-1491.

27. Li, S.Y., et al., *Efficacy of controlling night-time noise and activities to improve patients' sleep quality in a surgical intensive care unit.* J Clin Nurs, 2011. **20**(3-4): p. 396-407.

28. Locke, C.L. and D.S. Pope, *Assessment of Medical-Surgical Patients' Perception of Hospital Noises and Reported Ability to Rest.* Clin Nurse Spec, 2017. **31**(5): p. 261-267.

29. Lytle, J., C. Mwatha, and K.K. Davis, *Effect of lavender aromatherapy on vital signs and perceived quality of sleep in the intermediate care unit: a pilot study.* Am J Crit Care, 2014. **23**(1): p. 24-9.

30. Maidl, C.A., J.S. Leske, and A.E. Garcia, *The influence of "quiet time" for patients in critical care.* Clin Nurs Res, 2014. **23**(5): p. 544-59.

31. McDowell, J.A., et al., *A nonpharmacologic sleep protocol for hospitalized older patients.* J Am Geriatr Soc, 1998. **46**(6): p. 700-5.

32. Menger, J., et al., *Earplugs during the first night after cardiothoracic surgery may improve a fast-track protocol.* Minerva Anestesiol, 2018. **84**(1): p. 49-57.

33. Nooner, A.K., et al., *Using Relaxation and Guided Imagery to Address Pain, Fatigue, and Sleep Disturbances: A Pilot Study.* Clin J Oncol Nurs, 2016. **20**(5): p. 547-52.

34. Norton, C., et al., *Improving sleep for patients in acute hospitals.* Nurs Stand, 2015. **29**(28): p. 35-42.

35. Obanor, O.O., et al., *The Impact of Earplugs and Eye Masks on Sleep Quality in Surgical ICU Patients at Risk for Frequent Awakenings.* Crit Care Med, 2021. **49**(9): p. e822-e832.

36. Okkels, N., et al., *Lighting as an aid for recovery in hospitalized psychiatric patients: a randomized controlled effectiveness trial.* Nord J Psychiatry, 2020. **74**(2): p. 105-114.

37. Olson, D.M., et al., *Quiet time: a nursing intervention to promote sleep in neurocritical care units.* Am J Crit Care, 2001. **10**(2): p. 74-8.

38. Ong, T.L., et al., *Improving the Intensive Care Patient Experience With Virtual Reality-A Feasibility Study.* Crit Care Explor, 2020. **2**(6): p. e0122.

39. Ozlu, Z.K. and P. Bilican, *Effects of Aromatherapy Massage on the Sleep Quality and Physiological Parameters of Patients in a Surgical Intensive Care Unit.* Afr J Tradit Complement Altern Med, 2017. **14**(3): p. 83-88.

40. Patel, J., et al., *The effect of a multicomponent multidisciplinary bundle of interventions on sleep and delirium in medical and surgical intensive care patients.* Anaesthesia, 2014. **69**(6): p. 540-9.

41. Pati, D., et al., *The Impact of Simulated Nature on Patient Outcomes: A Study of Photographic Sky Compositions.* HERD, 2016. **9**(2): p. 36-51.

42. Pattison, H.M. and C.E. Robertson, *The effect of ward design on the well-being of post-operative patients.* J Adv Nurs, 1996. **23**(4): p. 820-6.

43. Richards, K.C., *Effect of a back massage and relaxation intervention on sleep in critically ill patients.* Am J Crit Care, 1998. **7**(4): p. 288-99.

44. Richardson, S., *Effects of relaxation and imagery on the sleep of critically ill adults.* Dimens Crit Care Nurs, 2003. **22**(4): p. 182-90.

45. Ryu, M.J., J.S. Park, and H. Park, *Effect of sleep-inducing music on sleep in persons with percutaneous transluminal coronary angiography in the cardiac care unit.* J Clin Nurs, 2012. **21**(5-6): p. 728-35.

46. Scarpa, M., et al., *Randomized clinical trial of psychological support and sleep adjuvant measures for postoperative sleep disturbance in patients undergoing oesophagectomy.* Br J Surg, 2017. **104**(10): p. 1307-1314.

47. Scotto, C.J., et al., *Earplugs improve patients' subjective experience of sleep in critical care.* Nurs Crit Care, 2009. **14**(4): p. 180-4.

48. Silvius-Byron, S.A., et al., *What is the best?: simple versus visitor restricted rest period.* J Nurs Adm, 2014. **44**(5): p. 291-7.

49. Smith, M.C., et al., *Outcomes of therapeutic massage for hospitalized cancer patients.* J Nurs Scholarsh, 2002. **34**(3): p. 257-62.

50. Spence, J., et al., *Nighttime noise issues that interrupt sleep after cardiac surgery.* J Nurs Care Qual, 2011. **26**(1): p. 88-95.

51. Su, C.P., et al., *A randomized controlled trial of the effects of listening to non-commercial music on quality of nocturnal sleep and relaxation indices in patients in medical intensive care unit.* J Adv Nurs, 2013. **69**(6): p. 1377-89.

52. Sweity, S., et al., *SleepSure: a pilot randomized-controlled trial to assess the effects of eye masks and earplugs on the quality of sleep for patients in hospital.* Clin Rehabil, 2019. **33**(2): p. 253-261.

53. Tas, D., et al., *Acupuncture as a complementary treatment for cancer patients receiving chemotherapy.* Asian Pacific Journal of Cancer Prevention: Apjcp. **15**(7): p. 3139-44.

54. Thomas, K.P., et al., *Sleep rounds: a multidisciplinary approach to optimize sleep quality and satisfaction in hospitalized patients.* J Hosp Med, 2012. **7**(6): p. 508-12.

55. van den Ende, E.S., et al., *Evaluation of Nonpharmacologic Interventions and Sleep Outcomes in Hospitalized Medical and Surgical Patients: A Nonrandomized Controlled Trial.* JAMA Netw Open, 2022. **5**(9): p. e2232623.

56. Van Rompaey, B., et al., *The effect of earplugs during the night on the onset of delirium and sleep perception: a randomized controlled trial in intensive care patients.* Crit Care, 2012. **16**(3): p. R73.

57. Vitinius, F., et al., *Feasibility of an interval, inspiration-triggered nocturnal odorant application by a novel device: a patient-blinded, randomised crossover, pilot trial on mood and sleep quality of depressed female inpatients.* Eur Arch Otorhinolaryngol, 2014. **271**(9): p. 2443-54.

58. Wakamura, T. and H. Tokura, *Influence of bright light during daytime on sleep parameters in hospitalized elderly patients.* J Physiol Anthropol Appl Human Sci, 2001. **20**(6): p. 345-51.

59. Yazdannik, A.R., et al., *The effect of earplugs and eye mask on patients' perceived sleep quality in intensive care unit.* Iran J Nurs Midwifery Res, 2014. **19**(6): p. 673-8.
